# Genomic Analysis of *Limosilactobacillus fermentum* ATCC 23271, a Potential Probiotic Strain with Anti-*Candida* Activity

**DOI:** 10.3390/jof7100794

**Published:** 2021-09-24

**Authors:** Camilla I. dos Santos, Carmem D. L. Campos, Wallace R. Nunes-Neto, Monique S. do Carmo, Flávio A. B. Nogueira, Rômulo M. Ferreira, Ennio P. S. Costa, Laoane F. Gonzaga, Jéssica M. M. Araújo, Joveliane M. Monteiro, Cinara Regina A. V. Monteiro, Fernanda S. Platner, Isabella F. S. Figueiredo, Rodrigo A. Holanda, Silvio G. Monteiro, Elizabeth S. Fernandes, Andrea S. Monteiro, Valério Monteiro-Neto

**Affiliations:** 1 Rede de Biodiversidade e Biotecnologia da Amazônia Legal, BIONORTE, São Luís 65055-310, MA, Brazil; camilla_itapary@hotmail.com (C.I.d.S.); wallaceneto2010@hotmail.com (W.R.N.-N.); enniocosta14@gmail.com (E.P.S.C.); 2 Laboratório de Microbiologia Aplicada, Universidade CEUMA, São Luís 65075-120, MA, Brazil; carmemcampos01@hotmail.com (C.D.L.C.); romulo.ferreira5@hotmail.com (R.M.F.); laoane_freitas@hotmail.com (L.F.G.); jessicamendesaraujo1@hotmail.com (J.M.M.A.); jovelianemello53@gmail.com (J.M.M.); rodrigo.holanda@ceuma.br (R.A.H.); andreasmont@gmail.com (A.S.M.); 3 Centro de Ciências Biológicas e da Saúde, Universidade Federal do Maranhão, São Luís 65080-805, MA, Brazil; carmo.monique@outlook.com (M.S.d.C.); augustofabn@gmail.com (F.A.B.N.); cinaraaragao@hotmail.com (C.R.A.V.M.); silvio.gm@ufma.br (S.G.M.); 4 Faculdades Pequeno Príncipe, FPP, Curitiba 80230-020, PR, Brazil; fsilvaplatner@gmail.com (F.S.P.); bellaafigueiredo@hotmail.com (I.F.S.F.); elizabeth.fernandes@pelepequenoprincipe.org.br (E.S.F.); 5 Instituto de Pesquisa Pelé Pequeno Príncipe, IPPPP, Curitiba 80250-060, PR, Brazil

**Keywords:** probiotic, genomic characterization, *Limosilactobacillus fermentum*, *Candida* infection

## Abstract

*Limosilactobacillus fermentum* (ATCC 23271) was originally isolated from the human intestine and has displayed antimicrobial activity, primarily against *Candida* species. Complete genome sequencing and comparative analyses were performed to elucidate the genetic basis underlying its probiotic potential. The ATCC 23271 genome was found to contain 2,193,335 bp, with 2123 protein-coding sequences. Phylogenetic analysis revealed that the ATCC 23271 strain shares 941 gene clusters with six other probiotic strains of *L. fermentum*. Putative genes known to confer probiotic properties have been identified in the genome, including genes related to adhesion, tolerance to acidic pH and bile salts, tolerance to oxidative stress, and metabolism and transport of sugars and other compounds. A search for bacteriocin genes revealed a sequence 48% similar to that of enterolysin A, a protein from *Enterococcus faecalis*. However, in vitro assays confirmed that the strain has inhibitory activity on the growth of *Candida* species and also interferes with their adhesion to HeLa cells. In silico analyses demonstrated a high probability of the protein with antimicrobial activity. Our data reveal the genome features of *L. fermentum* ATCC 23271, which may provide insight into its future use given the functional benefits, especially against *Candida* infections.

## 1. Introduction

*Candida* species are considered commensal microorganisms in humans [1]. Nevertheless, they can cause opportunistic infections, including some that are less severe and superficially located, and others of a life-threatening systemic nature, particularly in immunocompromised patients. Localized candidiasis mainly affects the mucosa of the oral cavity and vagina [2,3].

Azoles are commonly used to treat candidiasis [4]. These drugs are fungistatic, meaning that an efficient immune system is necessary to control the infection and resolve its symptoms [5]. Other classes of antifungals may also be indicated, particularly when the etiologic agent is resistant to azoles, including nystatin, amphotericin B, flucytosine or echinocandins [6,7]. Thus, there is a need for new effective therapeutic approaches for candidiasis.

According to The International Scientific Association for Probiotics and Prebiotics, probiotics are defined as “live microorganisms that, when administered in adequate amounts, confer a health benefit to the host” [8]. A probiotic formulation can be administered as a single microorganism or in association with different species. In addition, probiotics may be mixed with prebiotics to enhance their benefits. For human use, a probiotic should preferably be of human origin, safe, and free of genetic vectors capable of transferring antibiotic resistance and/or virulence genes. They must have the ability to survive in adverse host conditions (such as acidic pH, enzymes and bile acids) and to adhere to eukaryotic cells [9]. Probiotics can exhibit antagonism against microbial pathogens, stimulate the host immune system or confer other proven beneficial effects [8,10].

Health benefits have been reported in both human clinical trials and animal studies. The benefits include the reduction of antibiotic-associated diarrhea and rotavirus diarrhea [11], preventive and adjuvant therapy in the treatment of certain cancer types [12,13,14], immunological modulation [15], improved response to vaccination [16], adjuvant action in the treatment of *Helicobacter pylori* infections [17], relief of irritable bowel syndrome [18] and treatment and prevention of allergies [19], in addition to preventing type 1 diabetes in animal models [20].

The benefits produced by probiotics may be related to their ability to regulate the microbiota through competition for adhesion sites, as well as the production of soluble compounds, which can also have immunomodulatory or antimicrobial effects [21]. The modulation of the immune system by probiotics can result in enhanced antibody-mediated responses, reduced inflammation and increased phagocytosis, in addition to several other responses [22,23].

Many genera of bacteria (and yeasts) have been proposed as probiotics, but the most commonly used are *Lactobacillus* and *Bifidobacterium* species [24,25]. *Lactobacillus* is the most complex genus among lactic acid bacteria (LAB) and represents a defined group of Gram-positive, non-spore-forming rods or coccobacilli, which are fermentative, aerotolerant or anaerobic with a G + C content usually below 50 mol% [25]. The genus is included in the phylum Firmicutes and has recently been reclassified into 25 genera [26].

*Lactobacillus fermentum* has also undergone a change in its taxonomy, with its name being changed to *Limosilactobacillus fermentum* [26]. This species has been detected in human milk and feces, animal feces, plant tissues and dairy foods [27,28,29,30,31,32,33,34]. Some of the beneficial effects of this species, which are strain-specific, have already been described. These effects include reduction of cholesterol, prevention of community-acquired intestinal and upper respiratory infections, antioxidant potential, anti-aging action, anti-inflammatory activity, immune system stimulation and antimicrobial activity [27,28,29,30,32,33,35,36]. In our previous study, the *L. fermentum* ATCC 23271 strain, originally isolated from the human intestine, exhibited the capability to adhere to eukaryotic cells, mucin binding ability and inhibitory activities on the growth and cell adherence of genital pathogens, principally *Candida* species [37]. Thus, in order to gain further insight into the genetic basis of the probiotic potential of the *L. fermentum* ATCC 23271 strain, we sequenced its whole genome. We also compared its genome with the genomes of other *L. fermentum* strains in an attempt to identify its possible mechanisms of action.

## 2. Materials and Methods

### 2.1. Bacterial Strains and Growth Conditions

The following microorganisms were used in this study: *L. fermentum* ATCC 23271, enteroaggregative *Escherichia coli* (EAEC) 17.2, *E. faecalis* (ATCC 29212), *Salmonella enterica* subsp. *enterica* sorovar Enteritidis (ATCC 13076), *C. albicans* ATCC 90028, *C. albicans* SC 5314, *Candida krusei* ATCC 6258, *C. parapsilosis* ATCC 22019, *C. albicans* 44 (oral isolate), *C. albicans* CAS (vaginal isolate), *C. krusei* GJFD (vaginal isolate), *C. parapsilosis* FSG (oral isolate) and *C. parapsilosis* RCL (vaginal isolate). Probiotic properties were also evaluated against clinical *Candida* strains isolated from oral and vaginal cavities, belonging to the Culture Collection Sector of the Biology Laboratory of the Federal Institute of Education, Science and Technology of Maranhão. *L. fermentum* ATCC 23271 strains were cultivated in De Man, Rogosa, and Sharpe (MRS) agar and broth (Difco Laboratories, Detroit, MI, USA) under anaerobic conditions, while the other bacteria and yeasts were grown in brain heart infusion (BHI) agar/broth and Sabouraud agar, respectively (Difco Laboratories, Detroit, MI, USA), under aerobic conditions. All microorganisms were incubated at 37 °C for 24 h.

### 2.2. Whole-Genome Sequencing of Limosilactobacillus fermentum ATCC 23271

Genome sequencing of *L. fermentum* ATCC 23271 was performed by Neoprospecta using the Illumina HiSeq4000. Genomic DNA was extracted using the PureLink^®^ Genomic DNA Mini Kit (Invitrogen, Carlsbad, CA, USA) according to the manufacturer’s instructions. The complete genome was sequenced using the Illumina MiSeq paired library approach and prepared using the Nextera XT DNA Library Preparation Kit (Illumina, San Diego, CA, USA). The sequence readings were assembled using A5 software [38] and processed for adapter cutting, quality filtering and error correction to generate the contigs and scaffolds. In addition, CAP3 software [39] was used to improve the scaffolding assembly, cut low-quality regions and correct erroneous links between contigs. The pre-assembled genomic DNA sequences were annotated using the software tool Prokka [40].

### 2.3. Genome Analysis

The annotated sequences of the *L. fermentum* ATCC 23271 genome were analyzed using Rapid Annotation using Subsystem Technology (RAST) (available at https://rast.nmpdr.org/rast.cgi accessed on 10 January 2017). The Basic Local Alignment Search Tool (BLAST) was used to search for similarities in the protein databases of the National Center for Biotechnology Information (NCBI) and Universal Protein Resource. Phaster (https://phaster.ca/ accessed on 17 May 2021) was used to search for prophage-like clusters. Orthovenn (http://probes.pw.usda.gov/OrthoVenn accessed on 17 may 2021) was used to search for orthologous proteins common to the lineages *L. fermentum* 2760, *L. fermentum* DR 9, *L. fermentum* 3872, *L. fermentum* CECT 5716 and *L. fermentum* AGR 1485 [41]. To search for genes associated with bacteriocins, the BAGEL4 program (available at http://bagel4.molgenrug.nl/ accessed on 23 June 2020) was used, and a protein with similarity to another known bacteriocin was first analyzed using the Signal P 5.0 Server to identify the signal peptide cleavage region. The mature protein sequence was analyzed in the CAMP database using four machine learning algorithms, including Support Vector Machine, Random Forest, Artificial Neural Network and Discriminant Analysis, to predict its antimicrobial activity [42]. A phylogenetic tree was generated using the nucleotide sequence using the TYGS server (http://tygs.dsmz.de accessed on 3 February 2021) [43] using genomic sequences deposited in GenBank. To analyze evolutionary relationships between species, Mauve linked in Geneious software [44] was used. PathogenFinder v. 1.1 [45] was used to estimate the pathogenicity of the strain in human hosts. ResFinder v. 3.1 [46] and the Comprehensive Antibiotic Resistance Database (CARD) v. RGI 5.1.0, CARD 3.0.7 [47] were used to search for antimicrobial resistance genes. Virulence determinants were analyzed using VirulenceFinder v. 2.0 [48]. The rapid identification and annotation of prophage sequences within the genome of *L. fermentum* ATCC 23271 were performed using the phage search tool enhanced release (PHASTER) [49]. The CRISPR Finder tool was used to detect CRISPR direct repeats and spacers [50].

### 2.4. Antagonism Assay

Antagonism tests were performed using the overlay method [37]. *L. fermentum* ATCC 23271 grown on MRS agar was standardized at OD_600nm_ = 0.1, in MRS broth, and then 5 μL of the inoculum was spotted (about 0.9 cm) on MRS agar (Difco Laboratories, Detroit, MI, USA) and incubated for 48 h under the same conditions. After that period, a 2 mm layer of Muller–Hinton agar (Difco Laboratories) was added onto MRS agar, and the standard pathogen inoculum (1 × 10^8^ CFU/mL) was seeded. Petri dishes were incubated at 37 °C for 24 h under aerobic conditions, and subsequent zones of inhibition were measured.

### 2.5. Interference on Cell Adhesion

Cell adhesion experiments were performed was carried out with *Candida* spp. by using HeLa (ATCC CCL-2) cell line yeast, respectively [51], with minor modifications [37]. Cultivation was carried out in Dulbecco’s Modified Eagle’s Medium (DMEM, Sigma-Aldrich, Burlington, MA, USA) culture medium supplemented with 10% fetal bovine serum (FBS, Sigma-Aldrich) and 1% antibiotic and antimycotic solution, incubated in 5% CO_2_, at 37 °C in a humidity-controlled environment. The cells were seeded in 24-well plates at a concentration of 1 × 10^5^ cells/well and left to reach 80–90% confluence. Then, the microorganisms were added at concentrations of 1 × 10^6^ cells/well of pathogens and 1 × 10^8^ cells/well of *L. fermentum* ATCC 23271. The tests were performed in three different ways: by adding the two microorganisms at the same time (competition): first *L. fermentum* ATCC 23271 for 1 h and then pathogens (exclusion), or first pathogens for 1 h followed by *L. fermentum* ATCC 23271 (displacement). The plates were incubated for 4 h, and then dilution and plating were performed for CFU counts after incubation on Sabouraud agar.

### 2.6. Tolerance to Gastrointestinal Conditions

The tolerance of *L. fermentum* ATCC 23271 to acidic pH (pH = 2 and 4) and bile salts (0.5% and 1%, Oxgall, Sigma-Aldrich) was verified as described by Monteiro et al. [52]. Initially, 900 μL MRS (Difco Laboratories) was adjusted to pH 2 or 4 or supplemented with 0.5% or 1% (*w*/*v*) Oxgall (Sigma-Aldrich). The medium without modifications was used as a control. An aliquot of 100 μL of a 24 h culture of *L. fermentum* ATCC 23271, previously washed with PBS, was inoculated into tubes containing modified or unmodified MRS. After incubation at 37 °C for 3 h under anaerobic conditions, the percentage of viable bacteria was calculated by counting CFUs on MRS agar (Difco Laboratories).

### 2.7. Antibiotic Susceptibility Test

Antibiotic susceptibility assays were performed using the modified agar diffusion method, using commercial discs (Thermo Scientific^TM^ Oxoid^TM^) containing different antibiotics, including cefoxitin (30 μg), cefazolin (30 μg), chloramphenicol (30 μg), ciprofloxacin (5 μg), clindamycin (2 μg), erythromycin (15 μg), gentamicin (120 μg), linezolid (30 μg), nitrofurantoin (300 μg), oxacillin (1 μg), penicillin g (10 μg), rifampicin (5 μg), sulfazotrim (25 μg), tetracycline (30 μg), tigecillin (15 μg) and vancomycin (30 μg). *L. fermentum* ATCC 23271 overnight culture (37 °C in anaerobiosis) was standardized and inoculated on MRS agar medium, as previously described [53]. Subsequently, antimicrobial discs were placed, and the plates were incubated anaerobically at 37 °C for 24 h. The inhibition zone diameter of bacterial growth was measured (in mm), and susceptibility was assessed according to Charteris et al. [53].

### 2.8. Ethical Aspects

Clinical microbial strains were obtained from a previous research project approved by the Ethics Committee of CEUMA University (N° 2.519.446/2018/CEP-UNICEUMA).

### 2.9. Statistical Analysis

Data analysis was performed using GraphPad Prism Software 5.1 (GraphPad Software, San Diego, CA, USA). Student’s *t*-tests were performed to assess differences in the adhesion assays when the *Candida* strains were assayed alone or in the presence of *L. fermentum* ATCC 23271. All experiments were performed in triplicate on three independent days, and statistical significance was set at *p* < 0.05.

## 3. Results

### 3.1. General Genome Features and Comparative Analysis

To search for the genetic basis of the probiotic properties of interest, the genome of *L. fermentum* ATCC 23271 was sequenced. The genome was deposited in the NCBI database under the BioProject accession number PRJNA729474. Raw reads were deposited in the Sequence Read Archive (SRA) under the accession number SRX10856814. The assembled genome sequence was deposited in DDBJ/ENA/GenBank under the accession number GenBank: JAHBRU000000000.1.

Preliminary annotation data for the *L. fermentum* ATCC 23271 genome are listed in Table 1. The genome contains 2123 protein-coding sequences (CDS).

**Figure 1:**
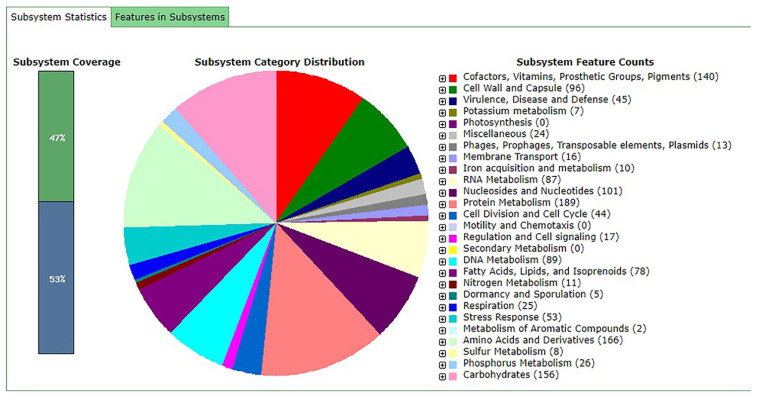
Categories of subsystems of the *L. fermentum* ATCC 23271 genome annotated by RAST.

The RAST analysis showed the presence of 2123 coding sequences distributed in 312 subsystems, of which 1614 were related to non-hypothetical proteins and 509 to hypothetical proteins. These genes were found to be associated with several subsystems (Figure 1). Through image analysis, it was seen that the subsystems with the highest number of genes are related to the production of cofactors, vitamins, prosthetic groups, pigments, protein metabolism, amino acids and carbohydrates.

**Figure 2:**
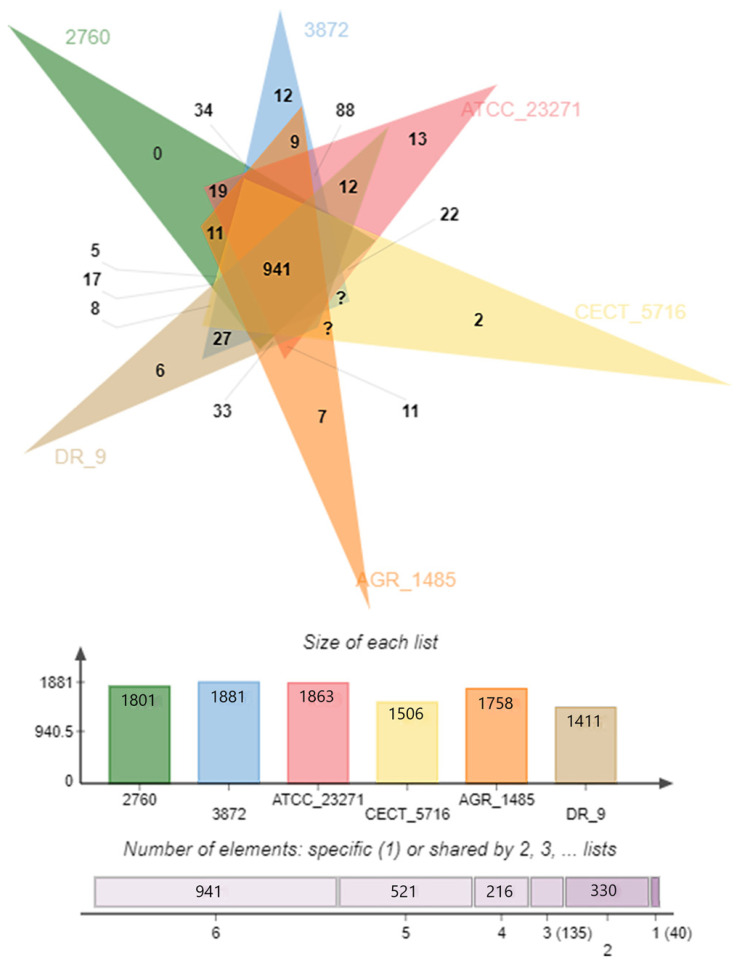
Venn diagram representing the groupings of orthologous genes shared between the following lineages of *L. fermentum*: ATCC 23271, 2760, 3872, CECT 5716, AGR 1485, and DR 9.

The genome of *L. fermentum* ATCC 23271 was compared with the whole genomes of other *L. fermentum* strains in GenBank using the OrthoVenn web platform. The species formed 2183 gene clusters, of which 1262 belonged to at least two species and 921 presented themselves in a single copy. It was also observed that *L. fermentum* ATCC 23271 shares 941 orthological clusters with all the other tested strains, and that it has 13 exclusive gene clusters (Figure 2).

**Figure 3:**
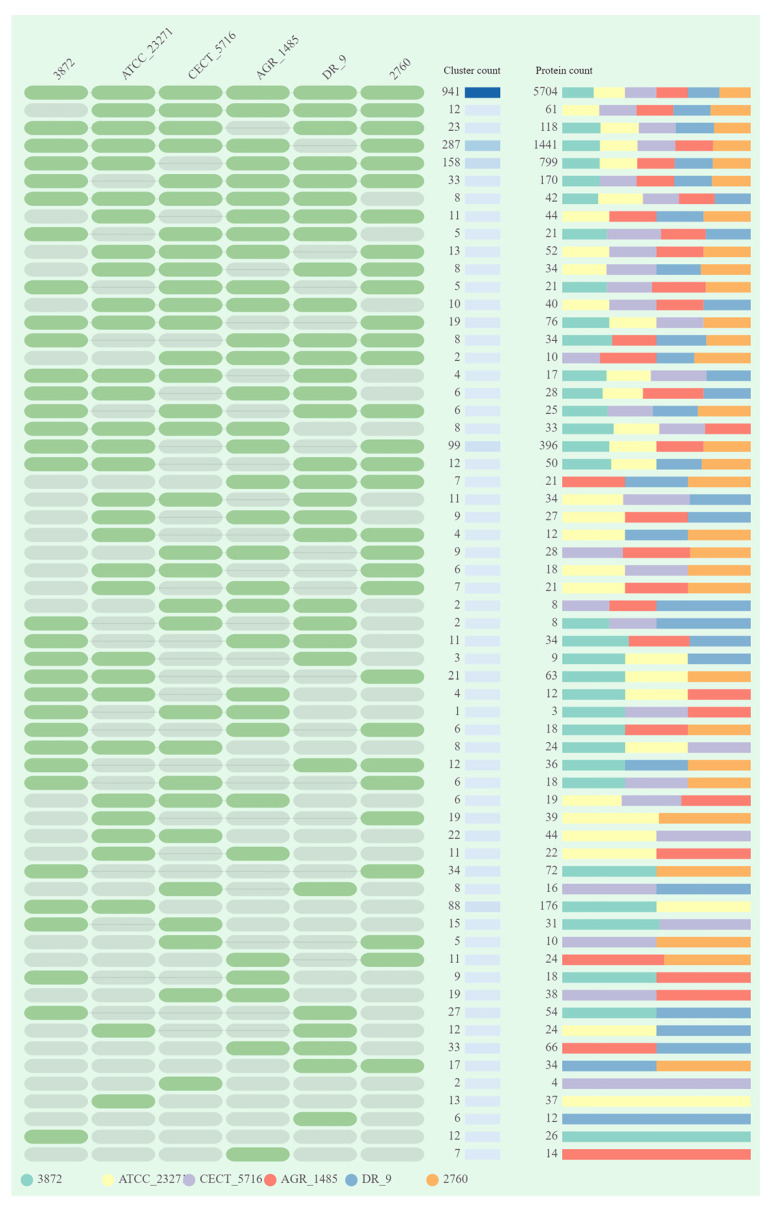
Overlays identified by the OrthoVenn 2 analysis of *L. fermentum* strains ATCC 23271, 2760, 3872, CECT 5716, AGR 1485 and DR 9.

The 941 shared clusters involved a total of 5704 proteins with each strain coding for approximately 16% of these proteins (Figure 3).

**Figure 4:**
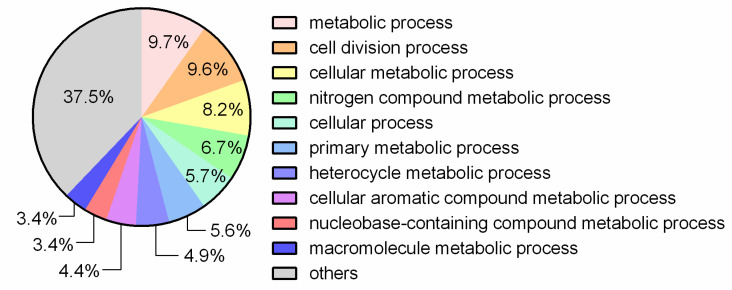
Processes related to proteins belonging to the clusters shared by the *L. fermentum* strains ATCC 23271, 2760, 3872, CECT 5716, AGR 1485 and DR 9.

Most proteins of these 941 clusters were found to be involved in the biological processes of chemical reactions and their pathways and cell division processes (Figure 4).

**Figure 5:**
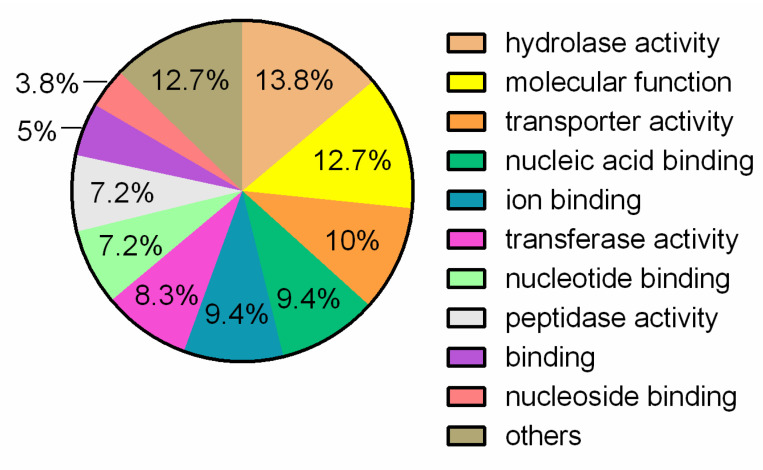
Molecular functions related to proteins belonging to the shared clusters by *L. fermentum* strains ATCC 23271, 2760, 3872, CECT 5716, AGR 1485 and DR 9.

With regard to molecular functions, 13.8% of the identified proteins are involved in hydrolase activity, 12.7% are related to a biochemical activity or are components of a larger process and 10% were responsible for the transport of substances inside, outside and between cells (Figure 5).

**Figure 6:**
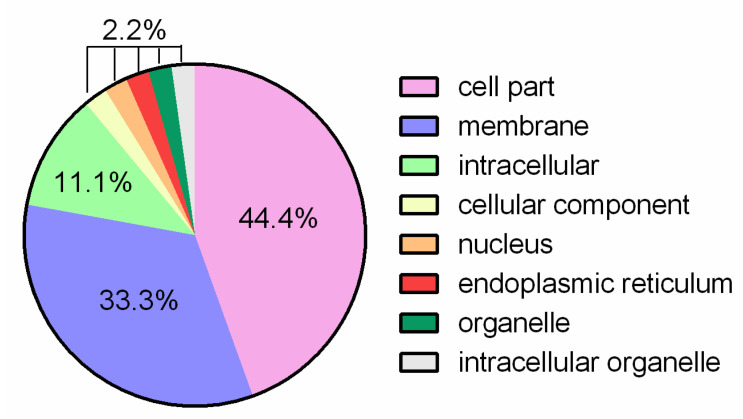
Cell components related to proteins belonging to the shared clusters of *L. fermentum* strains ATCC 23271, 2760, 3872, CECT 5716, AGR 1485 and DR 9.

The third group of proteins is related to the cellular components. In this group, most of the proteins comprised cell compartments (Figure 6).

**Figure 7:**
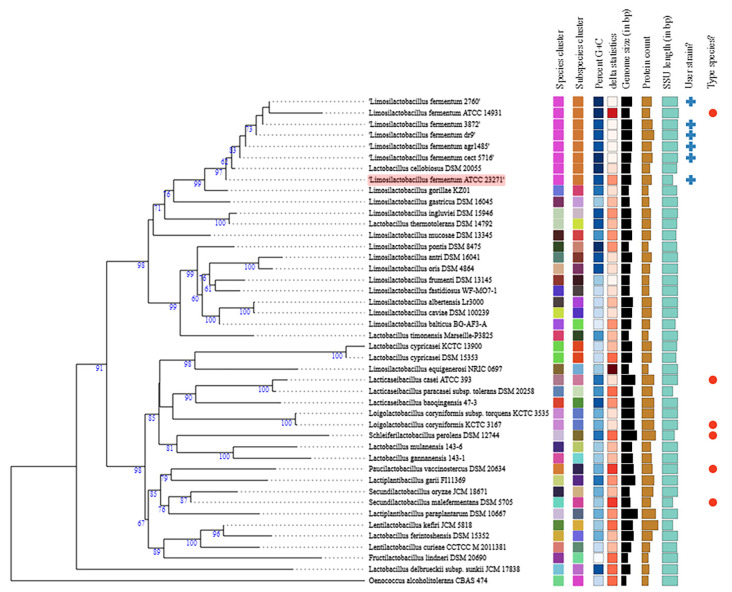
Phylogenetic tree of *Limosilactobacillus fermentum* ATCC 23271 and other bacterial genera available in the TYGS database. The tree was inferred using FastME 2.1.6.1 from ribosomal DNA. Branch lengths are scaled using the GBDP distance formula d5. The numbers above the branches are GBDP pseudo-bootstrap support values >60% of 100 replications, with an average branch support of 19.2%. The tree was rooted at the midpoint.

Using the reference genomes and other sequences available in the TYGS database, a phylogenetic tree was constructed (Figure 7). Phylogenetic tree analysis showed that the ATCC 23271 strain was the first species to appear during the evolutionary process. *Oenococcus alcoholitolerans* CBAS 474 was used as the outgroup and, therefore, the most distant on the evolutionary scale among all analyzed species and strains. *L. cellobiosus* DSM 20055 was also found to be inserted within the clade with other *L. fermentum* strains. The clade formed by *L. fermentum* had a 97% confidence level. *L. fermentum* ATCC 23271 was found to be more closely related to *L. cellobiosus* DSM 20055, within the clade, whereas the nearest outside species of the clade was *L. gorillae* KZ01.

Among the 13 exclusive clusters of *L. fermentum* ATCC 23271, OrthoVenn 2 analysis revealed 37 proteins that were most related to transposition. However, manual analysis showed only 16 ORFs, which included seven encoding transposases and six encoding hypothetical proteins, of which one sequence showed similarity to an *L. reuteri* hypothetical protein, two sequences that did not show similarity to any described protein and one encoded a chloride transporter, as evidenced by UniProt analysis.

### 3.2. Putative Genes Associated with Probiotic Properties

Genes for the following probiotic features were searched within the genome: tolerance to stress conditions, production of lactic acids, production of adhesion structures, production of antimicrobial peptides and safety for human use. Genomic analysis detected 21 genes encoding proteins that may be related to the ability to tolerate the secretion of digestive enzymes, bile salts and acidic pH. Among them, genes for sugar metabolism and production of L-lactic acid were also present in the genome (Table 2).

Eight genes were related to proteins involved in bacterial ability to adhere to and colonize eukaryotic cells, thus competing with pathogenic microorganisms for cell receptors, including: three related to the aggregation process, four related to the production of exopolysaccharides and a fibronectin-binding domain-containing protein (Table 3).

**Figure 8:**
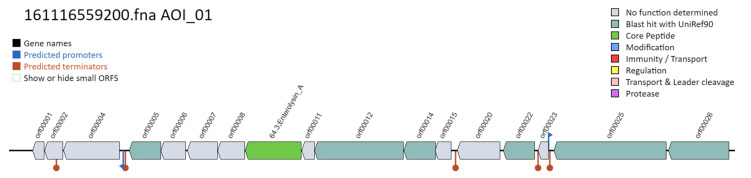
Prediction of a bacteriocin structure in the genome of *L. fermentum* ATCC 23271 strain.

A search for genes related to the production of bacteriocins was performed using the BAGEL4 program. A sequence 48% similar to that of enterolysin A, a protein of *Enterococcus faecalis,* was obtained. Figure 8 shows the position of the bacteriocin sequence in green.

After identifying the cleavage site of the translated bacteriocin protein sequence, using the SignalP 5.0 server, the mature protein was analyzed in silico to predict its antimicrobial activity by using four algorithms, including Support Vector Machine (SVM), Random Forest (RF), Artificial Neural Network (ANN) and Discriminant Analysis (DA). Of these four algorithms, three indicated a high probability (>0.9) of the peptide to present antimicrobial activity (Table 4).

In the genomic assessment of strain safety, the searches performed in both the ResFinder and CARD databases did not identify any genes encoding acquired drug resistance. However, the *L. fermentum* ATCC 23271 genome possessed 19 genes related to intrinsic resistance to antibiotics and other toxic compounds, including heavy metals such as cobalt, mercury, cadmium, copper and zinc, or represent potential targets for antimicrobial resistance, such as penicillin-binding protein, elongation factor G, DNA topoisomerase IV subunit B, topoisomerase IV subunit A, DNA topoisomerase (ATP-hydrolyzing) subunit B and DNA gyrase subunit A (Table 5).

In addition, the strain was predicted to be a non-human pathogen by the PathogenFinder tool hosted by the Centre for Genomic Epidemiology. The probability of being a human pathogen was calculated as 0.202, indicating a low probability for *L. fermentum* ATCC 23271 to present pathogenicity, and the estimated matched pathogenic families were 0. There was no hit for virulence determinants using the VirulenceFinder tool, also hosted by the Centre for Genomic Epidemiology. Two prophage regions were identified within the entire genome, and analysis using the PHASTER tool revealed that the sequences were incomplete. A search for the CRISPR-Cas sequence found two CDS putative sequences for the CRISPR sequences with the associated *cas* gene. These fragments occurred on contig identity NZ_JAHBRU010000076.1_1 (in the region between 17,626 and 18,783 bp) and NZ_JAHBRU010000115.1_1 (in the region between 850 and 3317 bp). The first identified CRISPR sequence contained 17 spacer genes and a 36 bp repeat consensus (GTCTTGGATGAGTGTCAGATCAGTAGTTCCGAGTAC), and the latter contained 40 spacer genes and a 28 bp repeat consensus (GGATCACCCCCATATACATGGGGAGCAC).

In addition, other putative genes were found in the genome of *L. fermentum* ATCC 23271 with important features including the following: (i) glutathione biosynthesis bifunctional protein (GshAB) and bifunctional glutamate-cysteine ligase/glutathione synthetase, which are involved in the biosynthesis of glutathione; (ii) peptide methionine sulfoxide reductases (MsrA and MsrB); (iii) free methionine-(R)-sulfoxide reductase; (iv) NADH peroxidase; and (v) thiol peroxidase. These are involved in the bacterial protection against oxidative stress [54,55,56,57].

### 3.3. Antagonism Activity

To confirm the inhibitory activity of the ATCC 23271 strain against *Candida* spp. and its low activity against bacteria [37], the overlay method was used as an antagonism assay. *L. fermentum* ATCC 23271 impaired the growth of *Candida* spp. with inhibition zones ranging from 13.5 ± 2.1 mm to 26.5 ± 2.1 mm. The only exceptions were two *Candida krusei* strains (ATCC 6258 and GJFD), which were not inhibited by the probiotic. Regarding the assays with bacterial strains, inhibition was observed only in the area immediately above the growth of the ATCC 23271 strain, that is, without the formation of large inhibition zones (Table 6).

### 3.4. Interference in Pathogen Cell Adhesion

**Figure 9:**
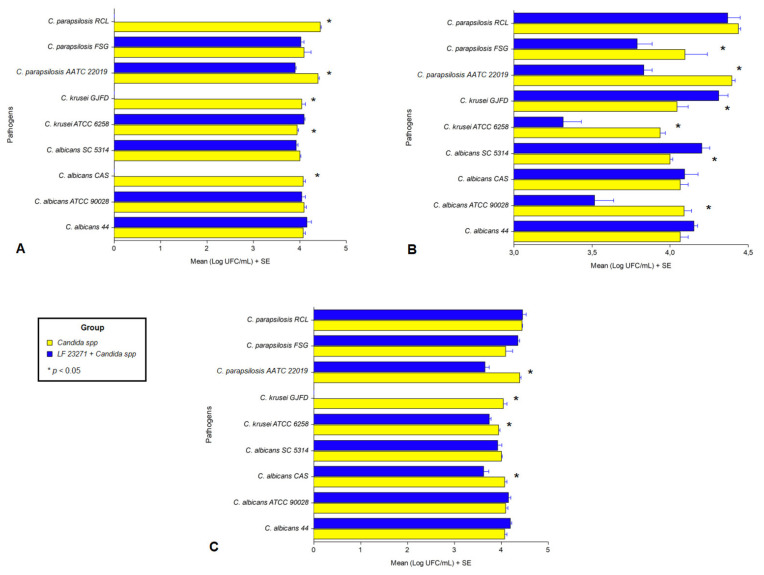
Inhibition of adhesion of *Candida* strains to eukaryotic cells by *L. fermentum* ATCC 23271 in three experiments: (**A**) competition, (**B**) exclusion and (**C**) displacement. * Represents the strains that had their adhesion to eukaryotic cells significantly influenced by *L. fermentum* ATCC 23271.

Due to the reduced inhibitory activity of *L. fermentum* on bacterial pathogens, interference assays on adhesion to HeLa cells were performed only with *Candida* strains. In the competition assay, three clinical strains (*C. albicans* CAS, *C. krusei* GJFD, and *C. parapsilosis* RCL) had their adherence to eukaryotic cells completely inhibited by *L. fermentum* ATCC 23271, an effect also noted for the reference strain *C. parapsilosis* ATCC 22019 (Figure 9A). In the exclusion experiment, on one hand, *L. fermentum* ATCC 23271 decreased the adhesion of *C. albicans* ATCC 90028, *C. krusei* ATCC 6258, *C. parapsilosis* ATCC 22019 and *C. parapsilosis* FSG to HeLa cells. On the other hand, *C. krusei* GJFD and *C. albicans* SC 5314 adhered more in the presence of *L. fermentum* ATCC 23271 (Figure 9B). In the displacement assay, *C. albicans* CAS, *C. krusei* GJFD, *C. krusei* ATCC 6258 and *C. parapsilosis* ATCC 22019 showed reduced adhesion to eukaryotic cells (Figure 9C).

### 3.5. Tolerance to Bile Salts and Acidic pH

To assess *L. fermentum* ATCC 23271 resistance to adverse host conditions, the bacterium was incubated in the presence of bile salts and acidic pH. The microorganism demonstrated a higher tolerance when cultivated at pH 4.0; however, it still had a survival rate greater than 60% when in a more acidic pH (pH 2.0). Exposure to bile salts did not affect *L. fermentum* growth. On the contrary, the highest survival rate was found following incubation with 0.5% bile salts (Table 7).

### 3.6. Antibiotic Susceptibility Assay

Antibiotic susceptibility was assessed by the disc diffusion method. *L. fermentum* ATCC 23271 was susceptible to most of the tested antibiotics. *L. fermentum* ATCC 23271 also exhibited moderate susceptibility to cefoxitin and norfloxacin and resistance to gentamycin, trimethoprim/sulfamethoxazole and vancomycin (Table 8).

## 4. Discussion

This study presents for the first time, a genomic analysis of *L. fermentum* ATCC 23271 in regards to important characteristics which confer a strain, a probiotic profile. In addition to its in vitro anti-*Candida* activity, different in silico functional analyses have revealed several protein-coding sequences associated with other probiotic properties of *L. fermentum* ATCC 23271, as well as with its safety for human use.

The *L. fermentum* ATCC 23271 strain was subjected to comparative analysis using the OrthoVenn program with others of the same species, which have previously reported beneficial effects. In this context, *L. fermentum* CECT 5716 strain has been extensively studied, and its probiotic properties include modulation of the host immune response [58], enhanced response to influenza vaccination in adults [59], reduction of *Staphylococcus* load in breast milk of lactating mothers [60], and reduced incidence of respiratory and gastrointestinal infections in infants [32]. The DR 9 strain has demonstrated antioxidant effects (via upregulation of oxoproline) and immunomodulatory effects in aging rats [61,62]; it also prevents telomere shortening in aging rats [63]. The *L. fermentum* 3872 strain is known for its potential for combating *Campylobacter jejuni* infections [30]. This strain also has a unique collagen-binding protein encoded by the *cbp* gene with five repetitive “B domains” (whereas the other *L. fermentum* strains only have four “B domains”) [64]. *L. fermentum* AGR1485 strain has the capacity to increase transepithelial electrical resistance (TEER) across Caco-2 cell layers, thus enhancing barrier integrity and protecting against infections by enteropathogens [65]. *L. fermentum* MCC 2760 has displayed several activities, including cholesterol reduction, high antioxidant potential, as well as anti-inflammatory actions [27,66]. Since *L. fermentum* ATCC 23271 shares 941 gene clusters with all those strains, it is possible that it also shares some of their probiotic properties.

Phylogenetic analysis showed that *L. fermentum* ATCC 23271 is part of the clade along with other strains of the same species, including *L. cellobiosus* DSM 20055. *L. cellobiosus* was first described by Rogosa et al. [67] and, like *L. fermentum*, is a heterofermentative *Lactobacillus* species. Both species share very similar phenotypic properties, and both belong to the subgenus ‘*Betabacterium*’ Orla-Jensen of *Lactobacillus* [68]. These authors support the idea that *L. cellobiosus* and *L. fermentum* should be united under the same name, with *L. fermentum* being the earlier synonym [68]. Previous studies have indicated a close relationship between *L. cellobiosus* and *Limosilactobacillus fermentum*; in fact, *L. cellobiosus* has now been reclassified as a biovar of *L. fermentum* [26]. The external lineage is *L. gorillae* KZ01, which is phylogenetically related to human-associated *L. fermentum*; however, it has not been found in humans yet and has been isolated from the feces of a captive western lowland gorilla (*Gorilla gorilla*) [69].

Concerning the tolerance to adverse conditions, the genome analysis showed that *L. fermentum* ATCC 23271 has several genes encoding proteins that are responsible for the resistance to acidic pH and bile salts. ATP synthases are some of the proteins involved mainly in acid tolerance, as they are associated with pH cytoplasmic regulation by ATP hydrolysis, which maintains pH homeostasis and protects cells from the damage induced by an acidic environment [70]. Glucose-6-phosphate isomerase is also involved in acid tolerance, acting as an acid shock protein [70]. All of these genes and others encoding proteins that promote acid and bile tolerance were detected in the genome of *L. fermentum* ATCC 23271 [71]. Phenotypic analysis also demonstrated its ability to survive in the presence of acidic pH and bile salts, corroborating its genetic background. In addition, *L. fermentum* ATCC 23271 contains putative genes encoding antioxidant enzymes, such as glutathione biosynthesis bifunctional protein, bifunctional glutamate-cysteine ligase, glutathione synthetase, peptide methionine sulfoxide reductases, free methionine-(R)-sulfoxide reductase, NADH peroxidase and thiol peroxidase. These enzymes (or their products) can protect cells against oxidative damage caused by reactive nitrogen intermediates and reactive oxygen species [55,72,73].

Additionally, other evidence shows that the enzyme methionine sulfoxide reductase (Msr) may also be involved in bacterial adhesion [74]. *L. fermentum* ATCC23271 has two types of this enzyme (MsrA and MsrB). In this regard, the ability to adhere to host tissues is an important attribute of probiotics [10]. *L. fermentum* ATCC 23271 presented nine genes encoding adhesion-related proteins, including exopolysaccharides (EPSs), which have different effects on probiotic adhesion to intestinal mucus, according to their different physicochemical and/or structural characteristics [75]. In addition, the gene that encodes the fibronectin-binding protein has also been found, and it is known to facilitate adhesion to the extracellular matrix of mammalian cells [76]. Both mechanisms could enable *L. fermentum* ATCC 23271 to adhere to host tissues and colonize the environment.

Competition for adhesion sites is an additional strategy used by probiotics to inhibit colonization by pathogens. In the present study, a strong ability to inhibit the adhesion of some *Candida* strains to eukaryotic cells was observed (mainly genital clinical isolates) in the competition and displacement assays. Heinemann et al. [77] showed that *L. fermentum* RC-14 is capable of releasing an active surface component, which can inhibit the adhesion of uropathogenic bacteria. In the same study, it was possible to purify a protein with anti-adhesive capacity against *Enterococcus faecalis* 1131. Another study showed that *L. fermentum* isolated from humans inhibits the adhesion of enteropathogens, such as that of *E. coli* to host cells, through an SAP protein [78]. The fact that some pathogens present greater adhesion in the presence of *L. fermentum* ATCC 23271 may be related to the production of aggregation proteins or EPS, as previously suggested [75,79,80].

Bacteriocins are small peptides secreted by many Gram-positive bacteria, with significant activity against distinct microorganisms [81]. A recent study demonstrated that SD11, an *L. fermentum*-derived bacteriocin, possesses anti-*Candida* activity [82]. Herein, genomic analysis of *L. fermentum* ATCC 23271 by the BAGEL 4 program showed the presence of a gene encoding a hypothetical protein with low similarity (48%) to enterolysin A. Interestingly, this bacteriocin is known to be produced by *Enterococcus faecalis* LMG 2333 and to inhibit some species of *Enterococcus*, *Lactobacillus*, *Lactococcus* and *Pediococcus* [83]. We also demonstrated that three algorithms indicated a high probability of this protein with antimicrobial activity. The evidence allows us to suggest that the antifungal activity observed in our study may be due to bacteriocins. However, further studies are necessary to determine the exact bacteriocins or any additional proteins involved in this activity.

Resistance to antibiotics is a concern because of the possibility of transferring the plasmid containing these genes to other pathogenic bacteria, making infections difficult to treat. The European Food Safety Authority (EFSA) recommends that bacterial strains harboring transferable antibiotic resistance genes should not be used as probiotics in animal feeds, fermented foods, and foods for human consumption [84]. Lactobacilli are susceptible to all protein synthesis inhibitors except aminoglycosides but are generally intrinsically resistant to quinolones, trimethoprim and sulphonamides, as well as, in the case of *L. fermentum*, also to glycopeptides [85]. All antibiotics to which *L. fermentum* ATCC 23271 showed resistance or moderate susceptibility indicate an intrinsic rather transferable resistance capacity.

*L. fermentum* ATCC 23271 presented two regions of prophages found in the genome characterized as incomplete, indicating that they were not functional. The identified transposases and other insertion sequences did not flank the resistance genes, further limiting their transferability. Furthermore, genome analysis revealed two regularly intercalated short palindromic repeat sequences (CRISPR) with the Cas gene and associated spacers. It has been reported that the presence of a CRISPR region may limit the spread of antimicrobial-resistant genes by inhibiting gene transfer pathways [86]. The CRISPR-Cas fragments work as a line of defense for the host strain against the insertion of extrachromosomal DNA molecules [50,86]. Therefore, their presence in the strain ATCC 23271 suggests a reduced probability of acquiring antimicrobial-resistant genes.

The protective role of *Lactobacillus* spp. against *Candida* spp. has been controversial, as the microorganism can be observed in high amounts in patients with *Candida* vaginitis [87]. In addition, many women with candidiasis may not have an altered microbiota [88]. These differences may be due to several factors including those inherent to the patient (such as age, immune status and symptoms) and factors related to the virulence properties of the *Candida* species causing the infection [89]. On the other hand, it has already been clearly demonstrated that a diversity of *Lactobacillus* spp. has antifungal effects [90,91,92]. Furthermore, only some strains of *Lactobacillus* can produce antimicrobial compounds in the amounts necessary for an antifungal activity, which possibly explains the failure of some vaginal lactobacilli to suppress colonization by *Candida*. In this context, our data indicate that *L. fermentum* ATCC 23271 may represent a potential strategy to prevent *Candida* colonization.

Genome sequence analysis can contribute to the understanding of the molecular basis of the probiotic functions of *L. fermentum* ATCC 23271. However, the genetic basis for growth and adhesion inhibitory activities against *Candida* need to be validated. Thus, gene knockout mutants of this strain are now being constructed to better assess the roles of the putative antimicrobial peptide and adhesin genes in these activities. The most notable feature is the number of genes associated with strain adhesion and survival under unfavorable conditions such as acidic pH, bile salts or even oxidative environments. Other interesting genomic components that represent some of the features that contribute to probiotic activity include many genes involved in sugar transport and metabolism, including oligosaccharides. One element that is part of the general characteristics of a probiotic is its safety for human use, which is assured by the absence of acquired antibiotic resistance and virulence genes, and the presence of gene loci associated with CRISPR/CRISPR (cas) and incomplete prophage regions.

## 5. Conclusions

The data from the present study demonstrate that *L. fermentum* ATCC 23271 is a probiotic candidate with anti-*Candida* activity. It can inhibit the growth of *C. albicans* and *C. parapsilosis*, in addition to being able to interfere with the adhesion of *Candida* spp. to host cells. Genomic analyses showed a variety of genes possibly associated with strain adhesion to host cells and molecules, tolerance to acidic pH, bile salts and oxidative stress as well as safety for human consumption. A limitation of the study is that the mechanism of growth inhibition of different *Candida* species is yet to be completely established. Although we have identified a probable protein with antifungal activity, we still need to validate this finding through studies with mutants, with synthetic peptides (whose sequences are derived from this protein) and also in animal experiments. An important aspect revealed by the comparative genomic analysis is that the strain shares many genes with other strains of the same species that have different probiotic properties. Nonetheless, our findings indicate a promising use of *L. fermentum* ATCC 23271 as an anti-*Candida* therapy.

## Figures and Tables

**Table 1:** Characteristics of the assembly of *L. fermentum* ATCC 23271 genome.

Attribute *	Indicators
Genome size (bp)	2,193,335
G + C content (%)	50.9
N50	33,843
L50	22
Number of contigs (with PEGs)	223
Number of subsystems	312
Prophage-like clusters	2
CDS	2387
CDS (with proteins)	2123
N° of RNAs	79
N° of tmRNA operons	1
N° of tRNA	55
N° of rRNA	21
N° of CRISPR loci	2

* Genome sequencing was performed using Illumina libraries designed using shotgun sequencing (Nextera). Genome annotation was performed automatically using the PROKKA tool.

**Table 2:** Genes possibly involved in acid and bile salt tolerance detected in the *L. fermentum* ATCC 23271 genome.

RAST/BLAST Description	Query Length	Accession Length	Query Cover	E Value	Per Ident	Accession
ATP synthase F0 sector subunit a/F0F1 ATP synthase subunit A	711 pb	236 aa	100%	7 × 10^−167^	100%	WP_003682740.1
ATP synthase F0 sector subunit b/F0F1 ATP synthase subunit B	507 pb	168 aa	100%	6 × 10^−116^	100%	WP_054173734.1
ATP synthase F0 sector subunit c/MULTISPECIES: F0F1 ATP synthase subunit C	213 pb	70 aa	100%	1 × 10^–38^	100%	WP_003682741.1
ATP synthase alpha chain/F0F1 ATP synthase subunit alpha	1539 pb	512 aa	100%	0.0	99.80%	WP_086439482.1
ATP synthase beta chain/F0F1 ATP synthase subunit beta	1422 pb	473 aa	100%	0.0	99.79%	WP_057194567.1
ATP synthase delta chain/F0F1 ATP synthase subunit delta	546 pb	181 aa	100%	3 × 10^–126^	99.45%	WP_057194565.1
ATP synthase gamma chain/F0F1 ATP synthase subunit gamma	936 pb	311 aa	100%	0.0	99.68%	WP_088460387.1
ATP synthase epsilon chain/	423 pb	140 aa	100%	3 × 10^–95^	99.29%	WP_003685876.1
L-lactate dehydrogenase (EC 1.1.1.27)/L-lactate dehydrogenase	954 bp	317 aa	100%	0.0	99.68%	WP_012391154.1
L-lactate dehydrogenase (EC 1.1.1.27)/L-lactate dehydrogenase	942 bp	313 aa	100%	0.0	100%	WP_138464682.1
L-lactate dehydrogenase (EC 1.1.1.27)/L-lactate dehydrogenase	933 bp	310 aa	100%	0.0	100%	WP_046948611.1
PTS system, cellobiose-specific IIC component/PTS system oligo-beta-mannoside-specific EIIC component	1311 bp	436 aa	100%	0.0	100%	QIX58800.1
ATP-dependent Clp protease ATP-binding subunit ClpX/ATP-dependent Clp protease ATP-binding subunit ClpX	1251 bp	416 aa	100%	0.0	99.52%	WP_096493701.1
Glucose-6-phosphate isomerase (EC 5.3.1.9)/glucose-6-phosphate isomerase	525 bp	174 aa	100%	1 × 10^–124^	99.43%	RGW51862.1
Glucose-6-phosphate isomerase (EC 5.3.1.9)/glucose-6-phosphate isomerase	1353 bp	450 aa	100%	0.0	99.56%	WP_021815746.1
GTP pyrophosphokinase (EC 2.7.6.5)/GTP pyrophosphokinase	612 bp	203 aa	100%	8 × 10^–150^	99.51%	WP_100184301.1
Pyruvate kinase/pyruvate kinase	1422 bp	473 aa	100%	0.0	100%	WP_003684953.1
Arginine/ornithine antiporter ArcD/Amino acid transporter	1419 bp	472 aa	100%	0.0	100%	AOR74635.1
Phosphoglycerate mutase/2,3-bisphosphoglycerate-dependent phosphoglycerate mutase	678 bp	225 aa	100%	4 × 10^–166^	100%	WP_004562727.1
Choloylglycine hydrolase/choloylglycine hydrolase family protein	978 bp	325 aa	100%	0.0	100%	WP_035436617.1
CTP synthase/CTP synthase	1602 bp	533 aa	100%	0.0	99.81%	WP_003684004.1

**Table 3:** Proteins potentially involved in the adhesion and aggregation properties of *L. fermentum* ATCC 23271 strain.

RAST/BLAST Description	Function	Query Length	Accession Length	Query Cover	E Value	Per Ident	Accession
Aggregation substance precursor	Aggregation	1842 pb	613 aa	99%	0.0	99.84%	AKM50933.1
LysM peptidoglycan-binding domain-containing protein		591 pb	196 aa	99%	9 × 10^–85^	100.00%	WP_021815732.1
LysM peptidoglycan-binding domain-containing protein		816 pb	315 aa	48%	1 × 10^–40^	100.00%	WP_168183590.1
Exopolysaccharide biosynthesis polyprenyl glycosylphosphotransferase	Exopolysaccharide production	666 pb	229 aa	99%	3 × 10^–159^	98.64%	WP_104877738.1
Exopolysaccharide biosynthesis protein		771 pb	256 aa	99%	0.0	99.22%	WP_163601282.1
CpsD/CapB family tyrosine-protein kinase		741 pb	247 aa	91%	3 × 10^–160^	99.12%	WP_062813522.1
Exopolysaccharide biosynthesis protein		771 pb	256 aa	99%	5 × 10^–174^	99.61%	KPH03198.1
Fibronectin-binding domain-containing protein	Adhesion	1692 pb	563 aa	99%	0.0	99.82%	WP_103205388.1

**Table 4:** Prediction of antimicrobial activity by using algorithms of CAMP_R3_ database.

Algorithms *	Results
SVM	1.000
DA	1.000
RF	0.957
ANN	NAMP

* Support Vector Machine, Discriminant Analysis and Random Forest algorithms report the result as a probability score (0 to 1); the Artificial Neural Network algorithm provides the results as either AMP (antimicrobial) or NAMP (not-antimicrobial).

**Table 5:** Putative genes involved in resistance to antibiotics and toxic compounds.

RAST/BLAST Description	Query Length	Accession Length	Query Cover	E Value	Per Ident	Accession
Penicillin-binding protein	1017	338	99%	0.0	99.41%	EQC60084.1
Class A beta-lactamase-related serine hydrolase	1020	339	99%	0.0	99.12%	MBD9348952.1
Cation diffusion facilitator family transporter	906	301	99%	0.0	100.00%	WP_015639412.1
Elongation factor G	2085	694	99%	0.0	99.86%	KPH03387.1
DNA topoisomerase IV subunit B	1998	665	99%	0.0	99.85%	WP_003683141.1
Topoisomerase IV subunit A	2478	825	99%	0.0	100.00%	BAG27240.1
MerR family transcriptional regulator	423	140	99%	1 × 10^–97^	100.00%	WP_003682036.1
Multidrug resistance protein MdtG	342	113	99%	2 × 10^–61^	99.12%	QIX57855.1
MFS transporter	123	160	97%	2 × 10^–17^	97.50%	WP_155762340.1
GTP-binding protein	1935	644	99%	0.0	100.00%	WP_112296957.1
Multidrug transporter	1227	413	99%	0.0	99.75%	AKM51464.1
DNA topoisomerase (ATP-hydrolyzing) subunit B	1950	649	99%	0.0	99.85%	WP_023465959.1
DNA gyrase subunit A	2511	836	99%	0.0	99.76%	WP_160229810.1
MerR family transcriptional regulator	423	151	99%	3 × 10^–98^	100.00%	CDI69999.1
Multidrug transporter MatE	1317	438	99%	0.0	99.77%	WP_042513988.1
Heavy metal translocating P-type ATPase	1929	642	99%	0.0	99.84%	WP_112297009.1
MATE family efflux transporter	1320	439	99%	0.0	97.95%	WP_135252410.1
FAD-dependent oxidoreductase	1350	449	99%	0.0	100.00%	WP_100184414.1
Cation transporter	552	207	99%	4 × 10^–98^	100.00%	WP_114684362.1

**Table 6:** Antagonism assay by the overlay method against *L. fermentum* ATCC 23271.

Pathogen	Inhibition Zone in mm ± SD *
*E. faecalis* (ATCC 29212)	In situ
*E. coli* enteroaggregative 17.2	In situ
*Salmonella enterica* (ATCC 13076)	In situ
*C. albicans* (ATCC 90028)	17 ± 1.41
*C. albicans* (SC 5314)	16.5 ± 1.12
*C. albicans* 44	14 ± 1.4
*C. albicans* CAS	20 ± 0
*C. krusei* (ATCC 6258)	−
*C. krusei* GJFD	−
*Candida parapsilosis* (ATCC 22019)	26.5 ± 2.1
*C. parapsilosis* FSG	13.5 ± 2.1
*C. parapsilosis* RCL	14.5 ± 0.7

* In situ inhibition occurred just above *L. fermentum* ATCC 23271 growth; − no inhibition.

**Table 7:** *L. fermentum* ATCC 23271 survival rate in the presence of acidic pH and bile salts.

Conditions	% of *L. fermentum* Survival (±SD) ^1^	*p*-Value ^2^
pH 2.0	60.88 ± 0.9569	0.0043
pH 4.0	105.0 ± 5909	
Bile salt 0.5%	109.7 ± 4434	0.0348
Bile salt 1%	94.13 ± 1244	

^1^ Data represent the survival percentage of *L. fermentum* ATCC 23271 after 180 min of exposure to different conditions compared to growth under normal conditions. ^2^ Statistical analysis was performed by Student’s *t*-test (*p* < 0.05).

**Table 8:** *L. fermentum* ATCC 23271 antimicrobial susceptibility by the disc diffusion method.

Antibiotics	*L. fermentum* ATCC 23271	
	Zone Inhibition in mm	Interpretation *
Cefazolin	22	Susceptible
Chloramphenicol	34	
Ciprofloxacin	21	
Clindamycin	31	
Erythromycin	39	
Linezolid	40	
Nitrofurantoin	30	
Rifampicin	34	
Penicillin G	41	
Tetracycline	38	
Tigecillin	40	
Cefoxitin	15	Moderately susceptible
Norfloxacin	14	
Gentamycin	12	Resistant
Sulfazotrim	0	
Vancomycin	0	

* Based on the criteria for the diameters of inhibition zones described by Charteris et al. [53].

## Data Availability

Sequence data used in this study were deposited at the National Center for Biotechnology Information (NCBI) as part of the BioProject PRJNA729474. Raw reads were deposited in the Sequence Read Archive (SRA) under the accession number SRX10856814. Whole Genome Shotgun assemblies have been deposited at DDBJ/ENA/GenBank under the accession number JAHBRU000000000.1.
